# Draft genome sequence of *Oryza sativa* elite indica cultivar RP Bio-226

**DOI:** 10.3389/fpls.2015.00896

**Published:** 2015-10-20

**Authors:** Mettu M. Reddy, Kandasamy Ulaganathan

**Affiliations:** Center for Plant Molecular Biology, Osmania UniversityHyderabad, India

**Keywords:** *Oryza sativa*, genome sequencing, yield, RP-Bio-226, nitrogen use efficiency

## Introduction

Rice cultivars show diversity in various factors influencing yield like nitrogen use efficiency, root development, and stress tolerance (Suryapriya et al., 2009; Singh et al., 2014; Begum et al., 2015; Mickelbart et al., 2015). For better understanding of the factors influencing yield of specific rice cultivars it is necessary to apply the high throughput genomic methods like whole genome sequencing, RNA-seq, smallRNA-seq, and ChIP-Seq. Improved Samba Mahsuri (RP Bio-226) is a bacterial leaf blight resistant indica rice cultivar developed through marker assisted selection from BPT5204 and SS1113. It is widely cultivated in Southern India because of its high yield, premium grain quality, and excellent cooking qualities. For understanding this cultivar in terms of nitrogen use efficiency recently we have sequenced the urea nutrition responsive transcriptome of this cultivar (Reddy and Ulaganathan, 2015). Further, with the aim of studying the genomic basis of yield of RP Bio-226 cultivar, we have sequenced the genome and analyzing it. Here we report the genomic dataset available publicly.

## Materials and Methods

### Plant Material

RP Bio-226 rice seeds (*O. sativa* sp. Indica) were surface- sterilized with 0.1% HgCl2 for 5 min and rinsed thoroughly with distilled water. These seeds were then transferred to Murashige and Skoog basal agar medium (Murashige and Skoog, 1962) for germination. Plants were grown in a culture room at 25°C, 60–80% relative humidity and 16 h (light)/8 h (dark) photo-period for 10 days. After this time, seedlings were harvested and used for genomic DNA isolation. The genomic DNA was isolated from the young leaves using cetyl trimethyl ammonium bromide (CTAB) method (Murray and Thompson, 1980). DNA concentration and purity were estimated using Nanodrop Spectrophotometer and Qubit Fluorometer.

### Library Preparation and Sequencing

For library preparation ∼3 μg of genomic DNA was sonicated using Covaris to obtain 200–500 bp fragment size. The size distribution was checked by running an aliquot of the sample on Agilent HS DNA Chip. The resulting fragmented DNA was cleaned up using High Prep PCR clean up beads. Fragmented DNA was subjected to a series of enzymatic reactions that repair ends, phosphorylate the fragments, and add a single ∼350–600 bp fragments was size selected on 2% low melting agarose gel and cleaned using MinElute column (QIAGEN). PCR (10 cycles) amplification of adapter ligated fragments was done and cleaned up using High Prep PCR beads. The prepared libraries were quantified using Qubit fluorometer and validated for quality by running an aliquot on High Sensitivity Bioanalyzer Chip (Agilent). Whole genome sequencing was carried out with Illumina_ Nextseq500 system (Illumina, San Diego, CA, USA). The raw read files in Fastq format were used for the genome assembly.

### Preprocessing and Genome Assembly

The preprocessing of raw reads was done with FastQC and the adapters were removed with Cut adapt (Andrews, 2010; Martin, 2011). After preprocessing the reads were aligned to the reference genome by using Bowtie2 (version 2.2.4) (Langmead and Salzberg, 2012). The indica rice 93-11 genome downloaded from BGI was used as the reference genome. Reference based assembly of the reads against the reference genome involved, indexing of the reference genome and alignment of reads to the reference and creation of SAM file. Samtools (version 0.1.18) and SnpEff (version 4.1) were used for further analysis (Li et al., 2009, 2012). SAM file was converted into binary BAM file, sorted and indexed by using the ‘view,’ ‘sort,’ and ‘index’ functions of Samtools. The quality of the assembly was checked by viewing the BAM file with ‘Bamview’ tool and used for variant calling. Samtools created a variation report from the Bowtie assembly with a mapping quality of >30 and read depth of >20 as cutoffs by using the mpileup function. It created a ‘bcf’ file which was converted into ‘vcf’ file with Bcftools. The duplicate variants were removed by varFilter in vcfutils. The consensus sequence was created with Samtools. Further annotation was done with snpEff (version 4.1) using the database built from the latest version of representative genes of RAP-DB (Sasaki et al., 2013). Gene prediction was carried out using Fgenesh program (Solovyev et al., 2006).

## Results

### Whole Genome Sequencing of RP Bio-226

The sequencing produced a total of 32,49,1164 paired-end reads of 151 bp length. These reads were checked for low quality and adapter contamination and the low quality reads and adapter sequences were removed using FastQC and Cutadapt tools. The remaining reads were assembled onto the reference genome, 93-11 indica rice genome, using Bowtie2 (Langmead and Salzberg, 2012). Over 90% reads were aligned to the reference genome and the overall coverage was estimated to be more than 20×. The Sam file produced by this reference based assembly was used for generating the variation (Single nucleotide polymorphism and indels) report using Samtools (Li et al., 2009). The variants reported by the Samtools were annotated with SnpEff tool (Li et al., 2012). The assembled genome size was estimated to be 347 MB and a total of 35,700 protein coding genes were predicted using Soft berry (Fgenesh) gene finding tool (Solovyev et al., 2006) (**Table 1**).

**Table 1:** *Oryza sativa* indica cultivar RP Bio-226 genome characteristics and resources.

Name	RP Bio-226 genome characteristic/resource
NCBI bioproject ID	PRJNA285384
NCBI biosample ID	SAMN03751782
NCBI SRA accession No.	SRP062659
NCBI genome accession numbers	CP012609-CP012620
Sequence type	Illumina_ Next seq500
Total number of reads	69,377,450
Read length	151
Overall coverage	20×
Mapped reads	90%
Estimated genome size	347MB
Predicted protein coding genes	35700
Genome download link	RP Bio-226 genome
Browse the genome	RP Bio-226 Gbrowse

### Direct Link to Deposited Data and Information to Users

The dataset submitted to NCBI include the assembled chromosomal sequences of *O. sativa* indica cultivar RP Bio-226 in Fasta format and the raw reads. The assembled chromosomal sequences in Fasta format and the raw reads can be accessed at NCBI with the following links CP012609–CP012620 and SRP062659, respectively. Users can download and use the data freely for research purpose only with acknowledgment to us and quoting this paper as reference to the data.

## Author Contributions

This work was planned by KU and execution was carried out jointly by both KU and MR.

## Conflict of Interest Statement

The authors declare that the research was conducted in the absence of any commercial or financial relationships that could be construed as a potential conflict of interest.
